# X-chromosome-linked miR-542-5p as a key regulator of sex disparity in rats with adjuvant-induced arthritis by promoting Th17 differentiation

**DOI:** 10.1186/s40364-025-00741-x

**Published:** 2025-03-01

**Authors:** Jiu Jie Yang, Zhi Li, Lin Na Wang, Bai Xiong Huang, Jerome P. L. Ng, Xiong Fei Xu, Yu Ping Wang, David Wei Zhang, Bo Qin, Ding Qi Zhang, Chang Liu, Wei Dan Luo, Betty Yuen Kwan Law, Hui Miao Wang, Meng Han Liu, Xiao Yun Yun, Joyce Tsz Wai Chan, Wan Yu Wu, Yi Ting Li, Peter Kam Fai Cheung, Man Chon Pou, Kat Sang Ha, Wang Fai Ao Ieong, Chi Hou Leong, Kit Ieng Leong, Chan Wang Lei, Lek Hang Cheang, Vincent Kam Wai Wong

**Affiliations:** 1 Dr. Neher’s Biophysics Laboratory for Innovative Drug Discovery, State Key Laboratory of Quality Research in Chinese Medicine, Macau University of Science and Technology, Macau SAR, China; 2 Macau Medical Science and Technology Research Association, Macao SAR, China; 3 Centro Hospitalar Conde de São Januário, Macau SAR, China

**Keywords:** Rheumatoid arthritis (RA), Sex disparity, Autoimmune disease, X chromosome, X-linked microRNA

## Abstract

**Background:**

Studies have indicated that X-linked microRNAs (miRNAs) play a role in the pathogenesis of rheumatoid arthritis (RA) and its gender-specific differences. However, research on specific miRNAs remains limited. This study aims to investigate the possible role of X-linked miR-542-5p in RA pathogenesis and gender differences.

**Methods:**

We investigated the impact of miR-542-5p on RA pathogenesis and gender differences by manipulating its expression in various rat models.

**Results:**

Our findings revealed a significant overexpression of miR-542-5p in RA patients compared with healthy individuals, with a notable gender difference among RA patients. In vivo experiments confirmed that upregulation of miR-542-5p could accelerate RA pathogenesis. Further analysis showed that the onset of adjuvant-induced arthritis (AIA) in rats exhibited significant gender differences, with more severe clinical phenotypes found in female rats. This may be attributed to their stronger immune responses and elevated levels of miR-542-5p. Subsequent in vitro and in vivo experiments demonstrated that miR-542-5p contributes to the regulation of gender differences in RA pathogenesis by promoting the differentiation of Th17 cells.

**Conclusions:**

This study offers new insights into the sex-specific nature of RA, suggesting X-linked miR-542-5p as a potential target for both diagnostic and therapeutic purposes. These findings lay the groundwork for the development of gender-specific therapeutic strategies for RA and underscore the importance of gender consideration in RA research.

**Graphical Abstract:**

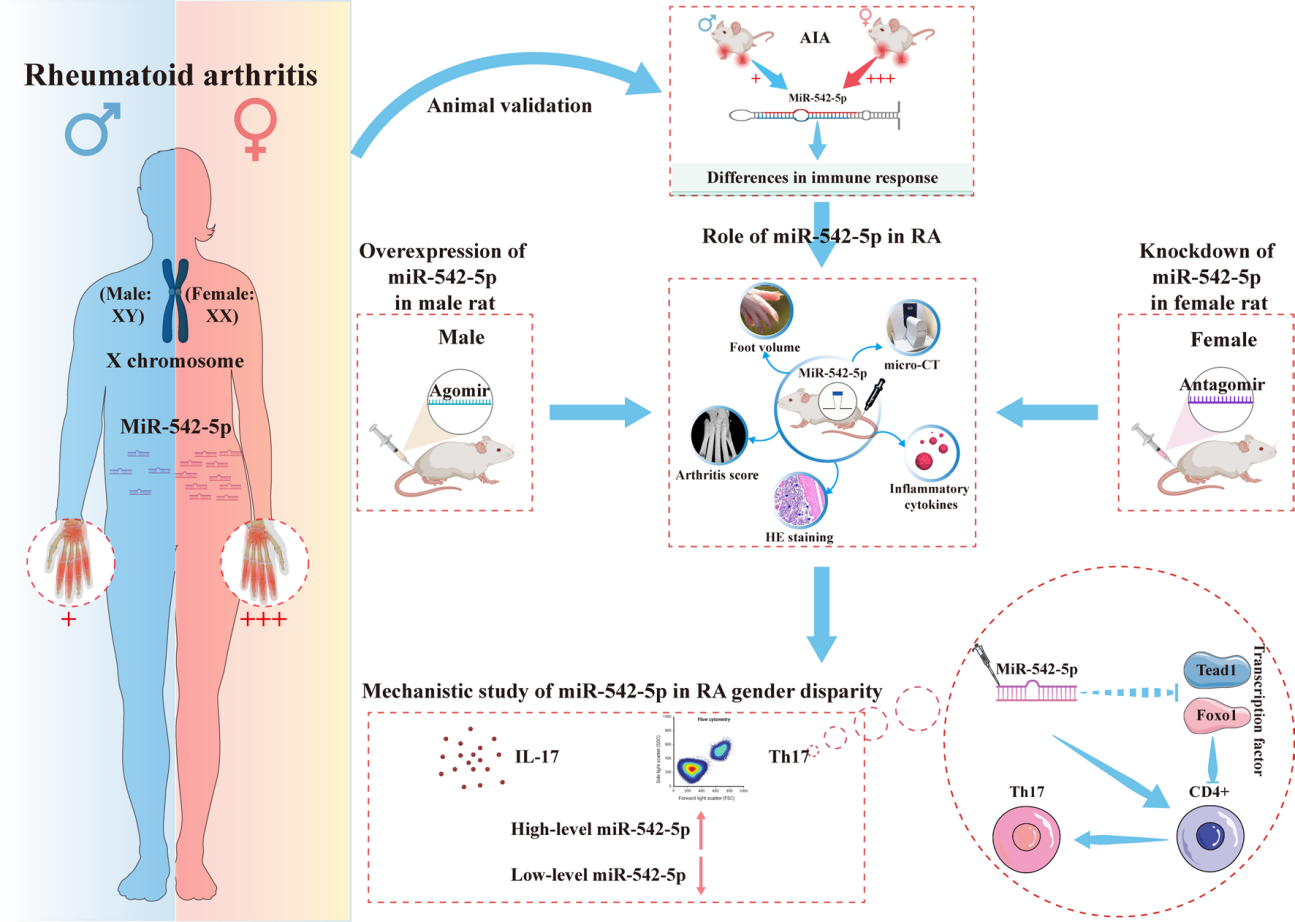

**Supplementary Information:**

The online version contains supplementary material available at 10.1186/s40364-025-00741-x.

## Introduction

Rheumatoid arthritis (RA), an autoimmune disease with multi-systemic inflammatory characteristics, prominently features joint swelling, deformity, and dysfunction [1]. Increasing evidence supports the existence of sex differences in RA, with females typically suffering more severe ramifications than males. Research findings have consistently confirmed that women are more predisposed than men to heightened clinical symptoms, such as joint deformities and swelling [2, 3]. Moreover, the prevalence rates and treatment responses vary between the female and male cohorts. In global, the prevalence of RA ranges from 0.5 to 1%, with a prevailing female-to-male ratio of approximately 3:1 [4].

Currently, gender differences in RA remain underexplored, yielding numerous hypotheses. One key theory is the substantial role of sex hormones, particularly evident in the impact of hormonal fluctuations during menopause and pregnancy on RA severity [5]. While the incidence in women is thrice that of men, pre-menopause, post-menopause and regional parity in incidence are observed. However, the influence of sex hormones alone appears insufficient to elucidate gender differences, given the significantly higher incidence of RA in prepubertal females [6]. Consequently, sex chromosomes play a critical role in RA. Studies on X chromosome-associated genes have emphasized the differences in immune responses, contributing to gender-based variations in RA etiopathogenesis [7]. In addition, ongoing research continues to explore the effects of epigenetic modifications, micro-chimerism, and skewed X chromosome inactivation on the incidence of RA in women [8, 9]. In our studies, we delve into the involvement of sex chromosomes in RA sexual dimorphisms.

The X chromosome consists of a large number of immune-related genes, possibly resulting in the observed sex differences in RA [10]. One of the two X chromosomes in females is subject to random inactivation (X chromosome inactivation, XCI) during embryogenesis in order to maintain a balanced gene dose between the sexes [11]. However, this inactivation is not fully complete, with approximately 15–23% of genes escaping this process, resulting in differential gene expression between the sexes [11]. For example, Johnston et al. reported that 5% of genes from the X chromosome, such as KDM5C, KDM6A, and DDX3X, exhibited significantly higher expression in females than males [11]. This inherent genetic asymmetry results in sex-specific differences in susceptibility to autoimmune diseases such as RA. In addition, it is noteworthy that the deregulation of certain genes from XCI, such as CD40L, IRAK-1 and TLR7, has been implicated in inflammatory responses and immune dysregulation [9], suggesting a potential link to the more severe clinical manifestations observed in female RA patients.

MicroRNAs (miRNAs) are small non-coding fragments of RNA (~ 22 bp) that have been implicated in the pathogenesis of many diseases, such as autoimmune diseases, cancers and inflammatory diseases, through the regulation of target gene expression [12]. Recent studies have shown that approximately 116 miRNAs are located on the X chromosome, a significantly higher number compared to only 2 miRNAs found on the Y chromosome [13]. These miRNAs are known as X-linked miRNAs that play important roles in immune and inflammatory responses, possibly leading to gender differences in the dynamics and extent of those responses [14, 15]. A recent study revealed that some X-linked miRNAs, such as miR-221, miR-222, and miR-92a, demonstrated significant sexual dimorphisms in individuals with RA [13]. Interestingly, all these miRNAs have already been reported either in RA or other inflammatory conditions. For example, lower expression of miR-221 suppresses the levels of pro-inflammatory cytokines and chemokines, which in turn inhibits RAFLS migration and invasion, and induces apoptosis [16]. Furthermore, miR-222 exerts arthroprotective effects in osteoarthritis (OA) by targeting HDAC-4 and inhibiting MMP-13 expression [17]. TLR-mediated downregulation of miR-92 in macrophages also induces an inflammatory response by targeting the MKK4/JNK/c-Jun pathway [18].

MiR-542-5p is one of the X-linked miRNAs, which plays a crucial role in immune regulation [19]. It has been shown that miR-542-5p can regulate the differentiation process of monocytes [20]. In fact, DNA damage, T-cell abnormalities, and mitochondrial homeostasis are important pathological mechanisms in RA [21–23], and studies suggest that miR-542-5p regulates these processes. For example, miR-542 can exacerbate the pathogenesis of Systemic Lupus Erythematosus (SLE) by regulating T-cell differentiation [24]. Moreover, miR-542-5p triggers the accumulation of reactive oxygen species and induces the decomposition of double-stranded DNA in transfected cells, thereby promoting cellular senescence [25]. A high level of miR-542-5p also induces abnormalities in mitochondrial function, thus exacerbating muscle weakness [26, 27]. Of note, miR-542-5p has been shown to escape from XCI [28], thus leading to variable expression between individuals. Currently, the role of miR-542-5p in gender bias of RA, and the mechanisms by which it may contribute, remain to be determined. In this study, we elucidated the role of X-linked miR-542-5p in the gender differences observed in RA, and proposed it as a potential therapeutic target for RA treatment and prevention, with a focus on gender-specific approaches.

## Materials and methods

### Clinical samples

All participants agreed and provided written informed consent. Peripheral blood samples were obtained from Centro Hospitalar Conde de Sao Januário (Macau, China). Epidemiological investigations and individual classifications were performed accordance with the principles of the European League Against Rheumatism. Clinical features of RA patients were shown in Table 1.

**Table 1:** The basic information of clinical samples obtained from RA patients and healthy individuals

	RA(*n* = 47)	Healthy(*n* = 61)
Age(years(mean(range)))	64(48–80)	58(35–74)
Sex(n(F/M))	47(30/17)	61(27/34)
ESR(mm/h(mean(range)))	15.6(3.4–4.1)	NA
RF(IU/mL(mean(range)))	233.4(105.5-399.5)	NA

### Quantitative analysis of mir-542-5p level

Total RNA, encompassing miRNAs, was extracted from human and rat peripheral blood mononuclear cells (PBMCs) by the mirVana™ miRNA Isolation Kit (Thermo Fisher), according to the manufacturer’s protocols. Subsequently, the quality and quantity of RNA samples were assessed using a NanoDrop 2000c Spectrophotometer (Thermo Fisher, USA). Reverse transcription and qRT-PCR for miR-542-5p were performed using a miDETECTA Track™ miRNA qRT-PCR Starter kit (RiboBio, Guangzhou, China) according to the manufacturer’s instructions on a BioRad IQ5 system. Each value was normalized to that of Rnu6. The relative gene expression levels were calculated using the 2^−ΔΔCt^ method. The primer sequences of miRNA were shown in Table 2.

**Table 2:** Primer sequences for miRNA quantification

Primer	Forward (5’-3’)	Reverse (5’-3’)
rno-miR-542-5p	CTCGGGGATCATCATGTCAC	GTGCAGGGTCCGAGGT
hsa-miR-542-5p	TCGGGGATCATCATGTC	GTGCAGGGTCCGAGGT

### Animal model

All experiments with rat were approved by the Animal Ethical Committee of the Department of Health and Supervision (Macao, China). The AIA rat model was established in Sprague Dawley (SD) rats weighing 130 ± 20 g (four per cage), sourced from ZHUHAI BESTEST BIO-TECH CO., LTD. Arthritis was induced by injecting complete Freund’s adjuvant (CFA) H37Ra (dried, heat-treated *Mycobacterium tuberculosis* strains dissolved in mineral oil at a concentration of 5 mg/ml, 0.1 mL) into the tail root of rats on Day 0. Simultaneously, rats in the healthy control group received injections of 0.1 mL saline solution. Arthritis scores and hind paw volumes were measured and recorded at three-day intervals until Day 27.

#### Model 1

SD rats were divided into four groups, each comprising five rats: (1) Healthy control(Ctrl); (2) AIA with agomir negative control (AIA/NC); (3) AIA + MTX (7.6 mg/kg, once a week (AIA/MTX)); (4) AIA + Ago-miR-542-5p (AIA/Agomir). AgomiR-542-5p (RiboBio) and the negative control (miR-Control, RiboBio) were administered via tail vein injection (20nmol/rat) on Day 0 and Day 10.

#### Model 2

All rats were stratified by sex into distinct cohorts: Females: (1) Ctrl, (2) AIA; Males: (1) Ctrl, (2) AIA.

#### Model 3

According to the gender of the rats, they were randomly divided into six groups (*n* = 5), with three groups of females and three groups of males. Females: (1) Healthy control (Ctrl), (2) AIA + antagomir negative control (AIA/NC), (3) AIA + miR-542-5p antagomir (AIA/Antagomir); Males: (1) Healthy control (Ctrl), (2) AIA + agomir negative control (AIA/NC), (3) AIA + miR-542-5p agomir (AIA/Agomir). Female AIA rats received interventions of antagomir negative control and antagomir on Day 0 and Day 10, respectively, at a dose of 100 nmol each time. Male AIA rats, received interventions of agomir negative control and agomir on Day 0 and Day 10, respectively, at a dose of 20 nmol each time.

### Flow cytometry analysis

Flow cytometry was used to isolate PBMCs from AIA rats when the experiment period are completed. The phenotype of T cells was validated using combinations anti-rat mAbs as follows: CD45 APC/Cy7, CD3 FITC, CD4 PerCP/Cy5, CD8APC, IL-17 A PE/cy7 (all purchased from BioLegend). Th17 cells were defined as CD4^+^IL-17 A^+^. For intracellular staining, PBMCs were stimulated with cell activation cocktail (BioLegend) for 4–5 h. Subsequently, cells were stained with surface antibodies, washed, fixed and permeabilized with Cyto-FastTM Fix/Perm Buffer Set (BioLegend) for 30 min. Intracellular labelling was performed with PE/cy7 conjugated anti-IL-17 A antibody for 1 h. FlowJo v10 software (TreeStar, Inc.) was used to analyze the flow cytometric data.

### Micro CT analysis

After euthanasia, the rats’ left hind paws were isolated and immersed in 4% paraformaldehyde (PFA) for fixation. The specimens were subjected to microCT analysis using a scanner (SkyScan 1176, Bruker, Belgium) with the following settings: resolution is 35 *µ*m resolution, tube parametersand is 85 *kV*, 385 *µ*A, exposure time is 65 ms, rotation step over 360° 0.7, and a 1 mm Al filter. NRecon software (Bruker-microCT, Belgium) was used to reconstruct micro-CT images. For analysis of the hind paw microstructure and disease-related parameters, CTvox software was utilized to examine the same anatomical region. The microCT scores were determined by averaging five indicators (trabecular number, bone mineral density, cortical mineral density, bone volume fraction and total porosity) with the scale categorized accordingly. The micro CT score was shown in Table 3.

**Table 3:** Micro CT score

Score	level
0-0.2	Mutilating
0.2–0.4	Several
0.4–0.6	Moderate
0.6–0.8	Mild
0.8-1.0	Normal

### Hematoxylin & eosin (H&E) staining

The knee tissues from each group were initially fixed in 4% paraformaldehyde for a minimum of 24 h, followed by dehydration and embedding in paraffin wax at 60 °C. Subsequently, 5 μm thick sections were prepared, dehydrated, deparaffinized, and rehydrated. Histological examination was conducted by staining the joint sections from each rat group using hematoxylin and eosin. The tissue images were then captured under an LED optical microscope (Leica DM2500).

### ELISA

The concentrations of cytokines were determined using LEGENDplex™ Rat Inflammatory Panel (Biolegend) or LEGENDplex™ Customer Panel (Biolegend) and cell sorter (BD Biosciences, USA). According to the manufacturer’s protocols, the detailed steps were as follows. 25 µL of assay buffer or matrix, 25 µL of diluted standard/sample and 25 µL of mixed beads were added to a 96-well plate. The plate was incubated for 2 h at room temperature with shaking at ~ 500 rpm. Then, each well was washed with 200 µL of 1x wash buffer for 2 times. Next, 25 µL of detection antibodies were added, incubated for 1 h at the room temperature with shaking at ~ 500 rpm. Subsequently, 25 µL of SA-PE was added and incubated for another 30 min under the same conditions. The wells were washed twice with 200 µL of 1x wash buffer using a vacuum manifold. After adding 150 µL of 1x wash buffer, the residue was re-suspended using a shaker for 1 min. Finally, the signal intensity of the microbeads was detected using a BD FACSAria III cell sorter (BD Biosciences, USA), and the data were analyzed using LEGENDplex software.

### Transfection and polarization of T helper 17 (Th17) cells

Naive CD4 ^+^ T cells were extracted from the splenocytes of SD rats using the Naive CD4 ^+^ T Cell Isolation Kit (Miltenyi Biotech), according to the manufacturer’s guidelines. MiR-542-5p agomir and corresponding negative control RNAs were sourced from RiboBio (Guangzhou, China). Oligonucleotides and Lipofectamine RNAiMAX transfection reagent (Invitrogen) were prepared as per the manufacturer’s guidelines and introduced into naive CD4 ^+^ T cells, which were stimulated with 2 µg/mL anti-CD3 (BD Pharmingen) on plates. Subsequently, naive CD4^+^ T cells were supplemented with TGF-β (1 ng/mL, Sinobioogical, China), IL-6 (5 ng/mL, Biolegend, USA), anti-IL-4 (10 µg/mL, BD Biosciences, USA), anti-CD28 (5 µg/mL, Bio X cell, USA), IL-1β (10 ng/ml, BD Biosciences, USA) and IL-23 (10 µg/mL, R&D Systems, USA) after 48 h transfection.

### Data and statistics

GraphPad Prism version 8.0 was employed for statistical analyses. Data presentation followed the format of mean values accompanied by either SD or SEM. Group disparities were evaluated employing the independent sample Student’s t-test or One-way ANOVA analysis. Correlation analyses were performed, with statistical significance denoted by a *P*-value less than 0.05.

## Results

### Expression of mir-542-5p in RA shows gender differences

**Fig. 1:**
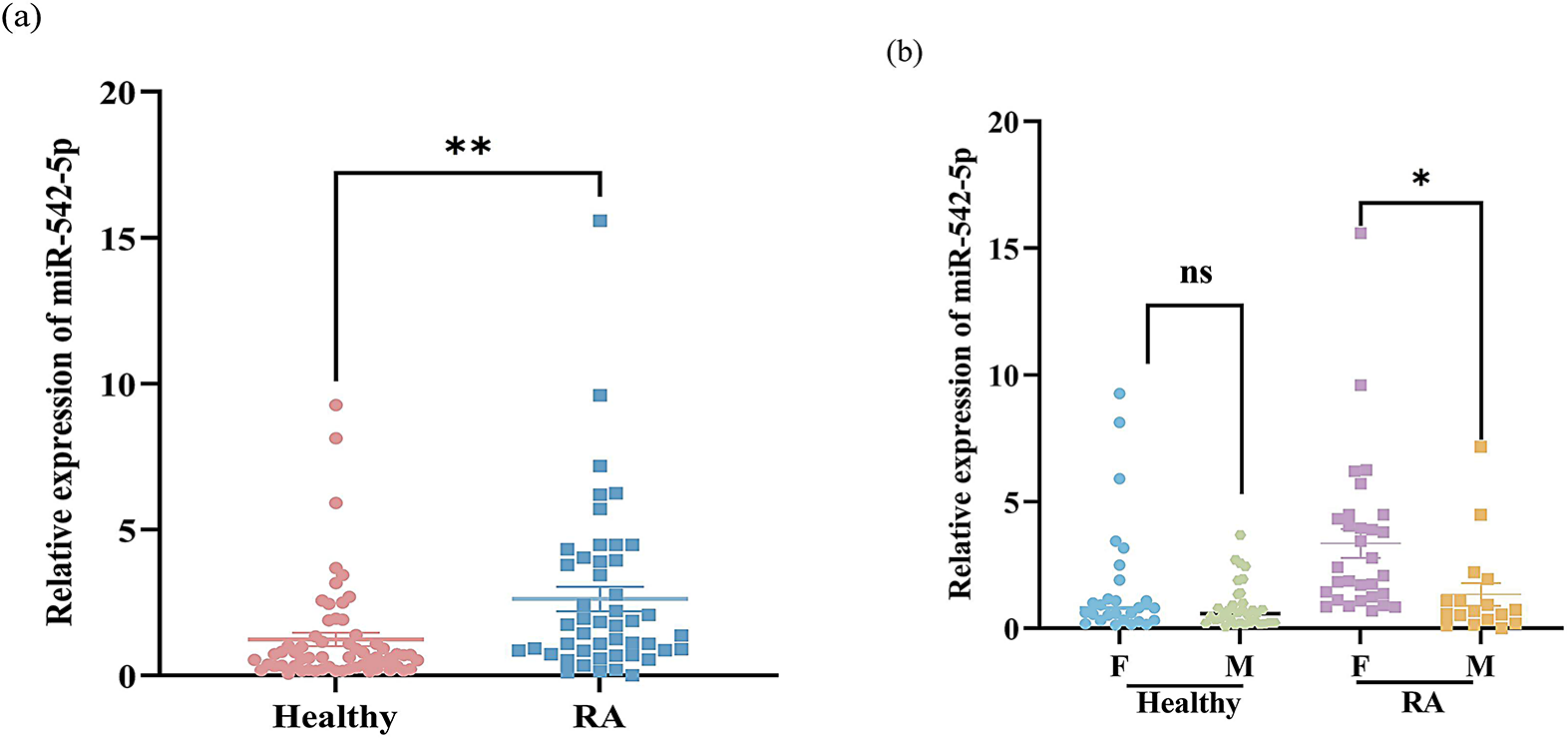
The expression level of miR-542-5p in the PBMCs of RA patients. PBMCs were isolated from the blood of patients with rheumatoid arthritis (RA) or healthy controls, and the expression level of miR-542-5p was quantified using real-time PCR. Endogenous RNU6 was used for normalization. (**a**) The expression levels of miR-542-5p in RA patients and healthy donors. The results are expressed as the means ± SEMs, with individual samples from 47 RA patients and 61 healthy subjects. ^**^P < 0.01, *t* test.(**b**) Patients in each group (RA and Healthy individual) were divided according to their gender. The results are expressed as the means ± SEMs of individual samples from 30 females with RA, 17 males with RA, 27 healthy females and 34 healthy males.,^*^P < 0.05, *t* test

Recent studies have shown that miR-542-5p expression is elevated in various types of tumors [29, 30]. Coincidently, elevated levels of miR-542-5p have also been observed in inflammatory diseases [31]. In conditions like SLE, increased miR-542-5p levels have been found to be associated with the intensification of disease onset [32]. To explore the correlation between miR-542-5p expression and the inflammatory manifestations of RA, we measured miR-542-5p expression in the peripheral blood of healthy volunteers and RA patients by real-time PCR. As shown in Fig. 1a, the peripheral blood expression of miR-542-5p in RA patients was significantly higher than that in healthy controls. In addition, miR-542-5p expression in the RA population strongly differed between genders, with higher expression in females (Fig. 1b). However, there was no obvious sex bias in the expression within the healthy population.

### Overexpression of miR-542-5p exacerbated disease severity in AIA rats

**Fig. 2:**
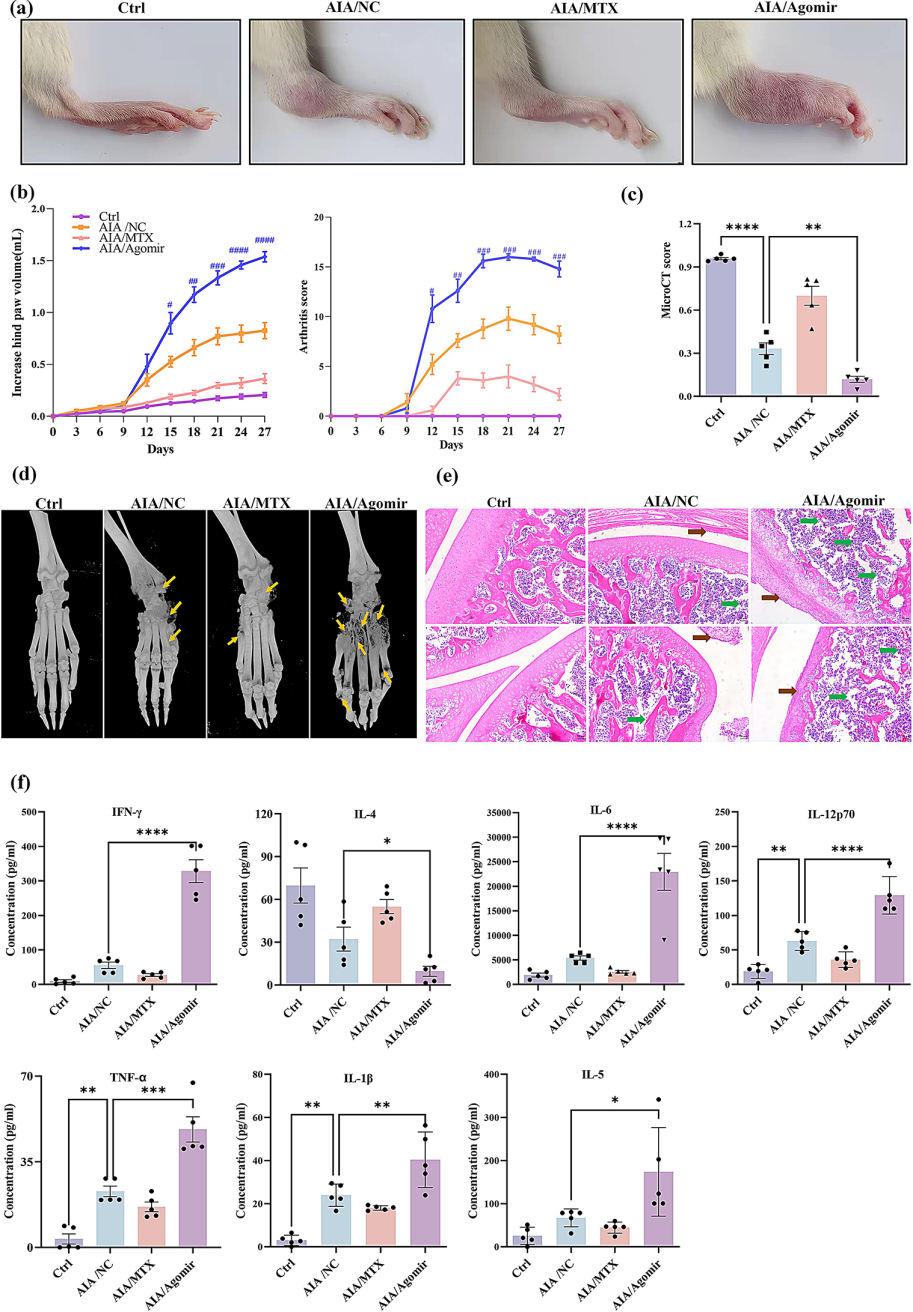
The exacerbating effect of the miR-542-5p agomir in AIA rats. (**a**) Representative images of the hind paws of miR-542-5p agomir-injected AIA rats on Day 27 after modeling. AIA rats were divided into three groups: the agomir-negative control (AIA/NC), MTX (7.6 mg/kg/week), and miR-542-5p agomir (AIA/Agomir) groups. (**b**) Changes in hind paw volume and arthritis scores were recorded every 3 days. A *t* test was utilized for comparison of the AIA/NC and AIA/Ago-miR-542-5p groups. The data are shown as the mean ± SEM (*n* = 5). ^#^*P* < 0.05, ^##^*P* < 0.01, ^###^*P* < 0.001. (**c**) The micro-CT scores of miR-542-5p agomir-injected AIA rats. A total of five indicators, including bone mineral density (BMD), bone volume fraction (BV/TV), cortical mineral density (TMD) in g/cm³, trabecular number (Tb.N) in mm⁻¹, and the percentage of total porosity, were analyzed to determine micro-CT scores. Smaller micro-CT scores indicate more pronounced bone destruction. The histograms are presented as the means ± SEMs (*n* = 5). ^**^P < 0.01, significantly different compared with the AIA/NC group, *t* test. (**d**) Representative micro-CT radiographic images of hind limb joint bone on day 27. Yellow arrows indicate bone erosion. (**e**) Representative images of H*&*E-stained knee tissues from each group (magnification, 50×). Green arrows indicate bone destruction, and brown arrows indicate proliferation of synovial tissue. (**f**) The serum concentrations of pro-inflammatory cytokines in each group were measured using a LEGENDplex™ Rat Inflammatory Panel. All the data are expressed as the means ± SEMs (*n* = 5). ^*^P < 0.05, ^**^P < 0.01, ^***^P < 0.001, significantly different compared with healthy controls or AIA/NCs, *t* test

On the basis of preliminary findings from clinical samples, miR-542-5p appears to play a pivotal role in the pathogenesis of RA. To elucidate the underlying mechanism, we established an AIA rat model to validate the influence of elevated miR-542-5p levels through tail vein injection of a miR-542-5p agomir, a stimulant known to mimic its miRNA expression. As shown in Fig. 2a and b, administration of miR-542-5p agomir exacerbated the arthritic phenotype in the hind paws of AIA rats, with higher paw volume and arthritis scores. Moreover, both microCT scores and imaging indicated markedly increased bone destruction in miR-542-5p agomir-injected AIA rats compared to untreated AIA rats (Fig. 2c and d). Additionally, H&E staining revealed more pronounced arthritic features in knee joints of agomir-treated AIA rats, characterized by synoviocyte hyperplasia and extensive bone erosion (Fig. 2e). The results of cytokines detection revealed a significant increase in the levels of proinflammatory factors, such as TNF-α, IL-1β, IL-6, IFN-γ, IL-5, and IL-12p70, in the miR-542-5p agomir-injected group. Conversely, the level of the anti-inflammatory factor IL-4 was significantly reduced in the agomir-treated group compared to the AIA group (Fig. 2f). These findings underscore that the overexpression of miR-542-5p expedites the progression of arthritis in AIA rats, indicating a prominent proinflammatory role in the context of RA.

### Female rats exhibit greater susceptibility to AIA induction than male rats

**Fig. 3:**
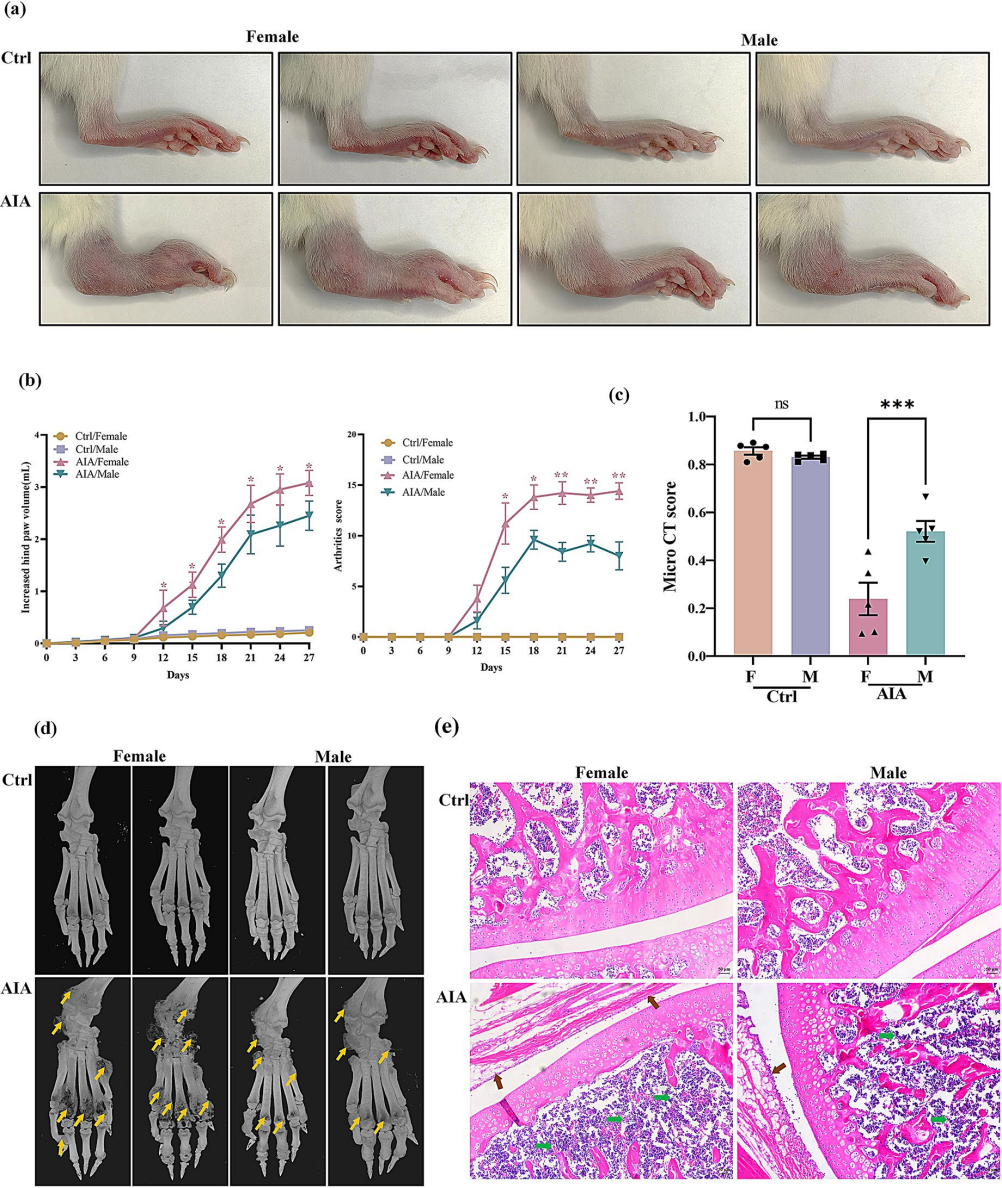
Female rats exhibit severe arthritis condition in AIA induction than male rats. (**a**) Representative images of the hind paws of female and male AIA rats on Day 27 after modeling. (**b**) Changes in hind paw volume and arthritis scores were recorded every 3 days. A *t* test was utilized for the comparison of female and male AIA rats. The data are shown as the means ± SEMs, *n* = 5. ^*^P < 0.05, ^**^P < 0.01. (**c**) The micro-CT scores indicated the gender differences in AIA rats. A total of five indicators, including BMD, BV/TV, and TMD in g/cm³,Tb. N in mm⁻¹ and the percentage of total porosity were combined and normalized to derive micro-CT scores. Smaller values indicate more pronounced bone destruction. The histograms are presented as the means ± SEMs (*n* = 5). ^**^P < 0.01, *t* test. (**d**) Representative micro-CT radiographic images of hind limb joint bone on Day 27. The yellow arrows indicate bone erosion. (**e**) Representative images of H&E-stained knee joint tissues from each group (magnification, 50×). Green arrows indicate bone erosion, and brown arrows indicate the proliferation of synovial tissues

**Fig. 4:**
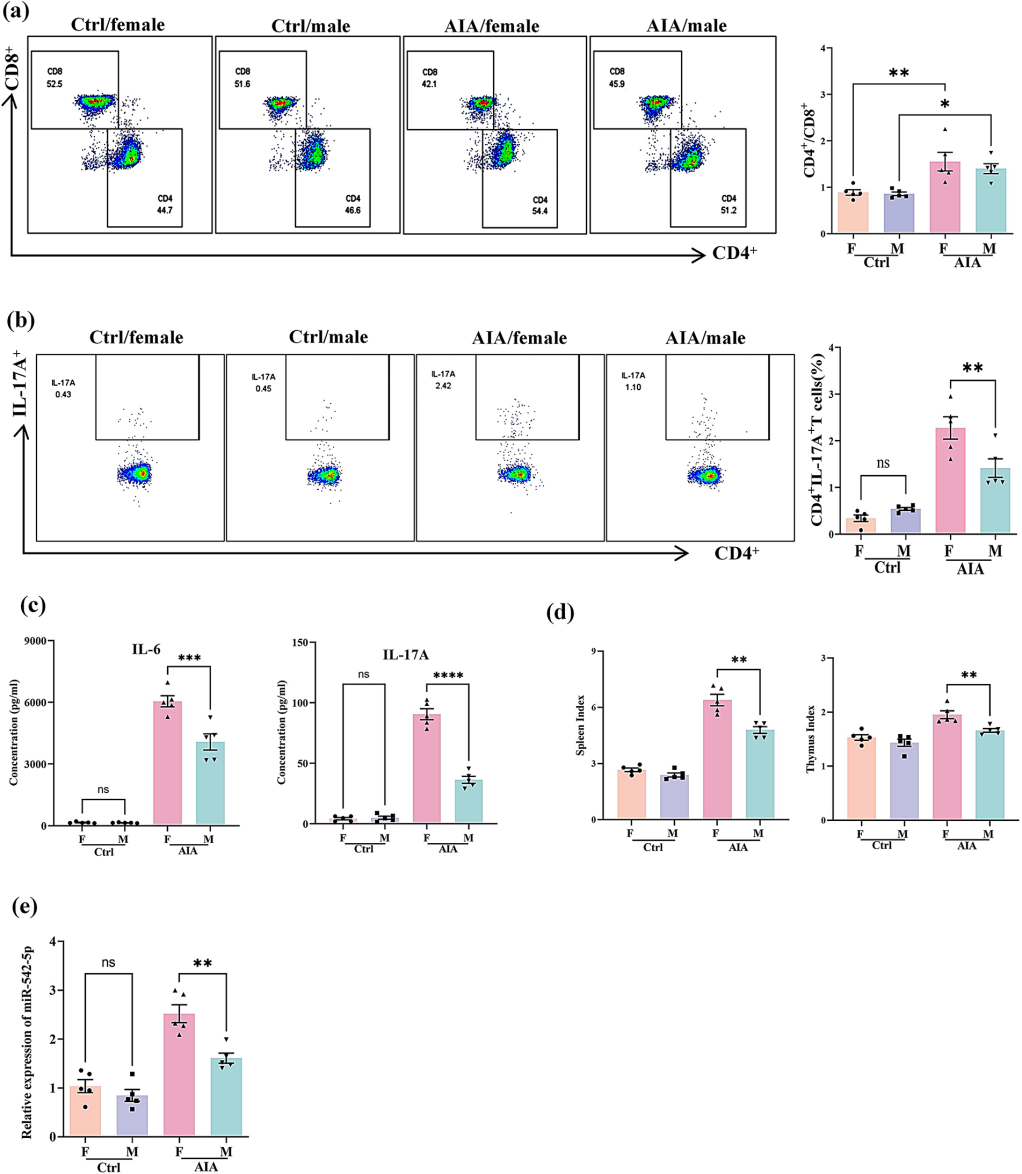
Gender differences affect autoimmune responses in AIA rats. (**a**) Representative flow cytometry plots depicting the proportions of CD4^+^ and CD8^+^ T lymphocytes within the CD3^+^ T lymphocyte subset. The bar chart shows the quantitative analysis of CD4^+^/CD8^+^ T-cell ratio. (**b**) The frequency of IL-17 A^+^ T cells within the CD4^+^ T lymphocyte population. The bar chart shows the quantitative analysis of the proportion of IL-17 A^+^ T cells among CD4^+^ T lymphocytes. ^*^P < 0.05, ^**^P < 0.01, *t* test. (**c**) The serum concentrations of pro-inflammatory cytokines (IL-6 and IL-17 A) were measured using a LEGENDplex™ Customer Panel. The data are presented as the means ± SEMs (*n* ≥ 5). ^***^P < 0.001, ^****^P < 0.0001, *t* test. (**d**) The spleen and thymus indices are expressed as the ratio of the wet weights of the spleen and thymus to the body weight (mg/g). The data are expressed as the mean ± SEM (*n* = 5). ^**^P < 0.01, *t* test. (**e**) Differential expression of miR-542-5p in AIA rats across genders. The expression level of miR-542-5p in peripheral blood was quantitatively measured using real-time PCR. The miRNA expression data were normalized to the reference gene RNU6 by employing the 2^−ΔΔCT^ method for relative quantification. Statistical analysis revealed a significant difference (^**^P < 0.01, *t* test) between the experimental groups

To investigate gender differences in RA development and the innate immune response, the clinical phenotypes and corresponding immune indices in an AIA rat model were investigated. As shown in Fig. 3a and d, female AIA rats presented more severe swelling, erythema, and bone destruction as indicated by their arthritic and microCT scores. Histopathological analysis further revealed that female AIA rats displayed more pronounced arthritic features, including synovial cell hyperplasia with neutrophilic infiltration and bone destruction (Fig. 3e). We then investigated the immune responses in rats, and observed a more severe immune reaction in female AIA rats than in male AIA rats, as anticipated. T-cell analysis revealed a higher CD4^+^/CD8^+^ T-cell ratio in AIA rats, with no notable gender differences (Fig. 4a). Notably, female AIA rats presented a higher proportion of Th17 cells than male AIA rats (Fig. 4b). Elevated serum concentrations of IL-17 A and IL-6 further confirmed the abnormal increase in Th17 cell differentiation in female AIA rats (Fig. 4c). As shown in Fig. 4d, spleen and thymus indices were significantly elevated in female AIA rats compared with male AIA rats, indicating the significant increase in autoimmune activity. Furthermore, we detected higher levels of miR-542-5p expression in female AIA rats than in male AIA rats (Fig. 4e). These findings suggest that female AIA rats exhibit a more severe RA clinical phenotype, potentially correlated with the elevated miR-542-5p expression and increased Th17 cell differentiation.

### Immuno-inflammatory role of miR-542-5p in gender differences of AIA rats

**Fig. 5:**
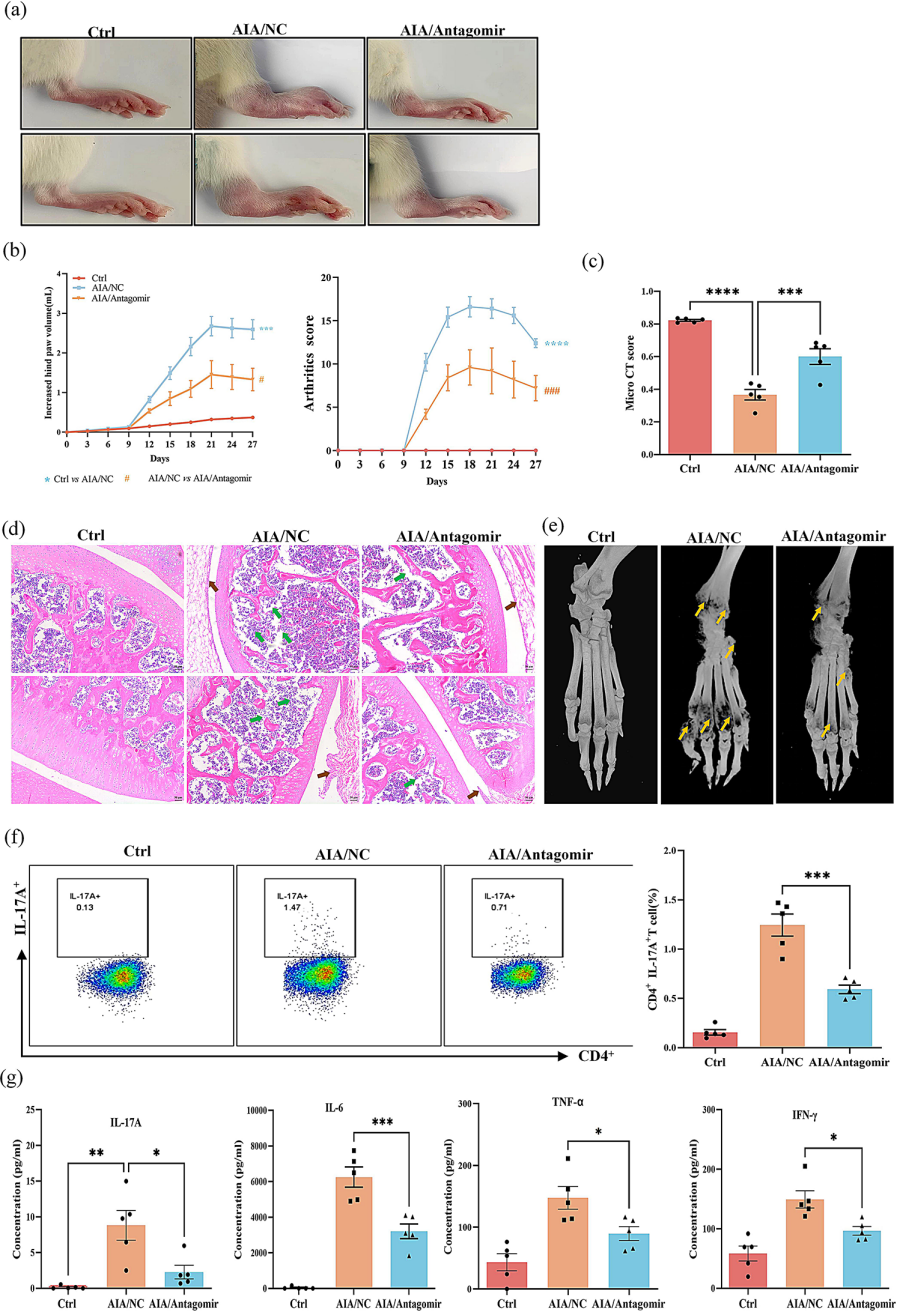
Effect of the miR-542-5p antagomir on the clinical phenotype and regulation of Th17 cells in female AIA rats. (**a**) Representative images of hind paws of female rats treated with miR-542-5p antagomir on day 27. (**b**) Changes in hind paw volume and arthritis score of female AIA rats treated with miR-542-5p antagomir were determined every three days for 27 days after modeling. A *t* test was utilized for comparisons between the AIA/NC and AIA/antagomir groups. The results are expressed as the mean ± SEM, *n* = 5. ^***^P < 0.001, ^****^P < 0.0001, ^#^*P* < 0.05, ^###^*P* < 0.001. (**c**) Micro CT score of AIA rats. The data are expressed as the mean ± SEM (*n* = 5). ^***^P < 0.001, ^****^P < 0.0001, vs. the AIA/NC group, *t* test. (**d**) Representative images of H&E-stained knee tissues from each rat group (magnification, 50×). Green arrows indicate bone destruction, and brown arrows indicate proliferation of synovial tissues. (**e**) Representative images of 3D reconstructions of micro-CT scans of rat hind limbs. The yellow arrows indicate bone erosion. (**f**) Representative flow cytometry plot images illustrating the percentages of IL-17 A^+^ T cells among the CD4^+^ T lymphocyte population. Bar charts indicate the proportions of Th17 cells among the CD4^+^ lymphocyte population. The data are presented as the means ± SEMs (*n* = 5), ^***^P < 0.001, compared with AIA/NC group, *t* test. (**g**) The concentrations of pro-inflammatory cytokines IL-17 A, IL-6, IFN-γ and TNF-α in the serum of female rats were detected by a LEGEND plex rat inflammation panel assay. All the data are shown as the means ± SEMs (*n* = 5). Statistical significance was calculated by *t* tests, ^*^P < 0.05, ^**^P < 0.01, ^***^P < 0.001, compared with the healthy control or AIA/NC group

To investigate whether the observed gender disparities in RA are attributed to differential miR-542-5p expression, we suppressed miR-542-5p in female AIA rats via tail vein injection of antagomir. A significant decrease in miR-542-5p expression was observed in the AIA/anti-miR-542-5p group relative to the AIA group (Fig. S1a). As demonstrated in Fig. 5a-e, downregulation of miR-542-5p alleviated the clinical phenotype of RA in female AIA rats, such as reductions in paw swelling, bone destruction and synovial hyperplasia. Notably, both the proportion of Th17 cells and serum concentrated of IL-17 A were markedly decreased in antagomir-treated female AIA rats (Fig. 5f-g). Additionally, the suppression of inflammatory cytokines releases further validated the inhibitory effect of miR-542-5p downregulation on immune responses in AIA rats (Fig. 5g). The immune organs index was significantly decreased in AIA rats after down-regulating miR-542-5p (Fig. S1b).

**Fig. 6:**
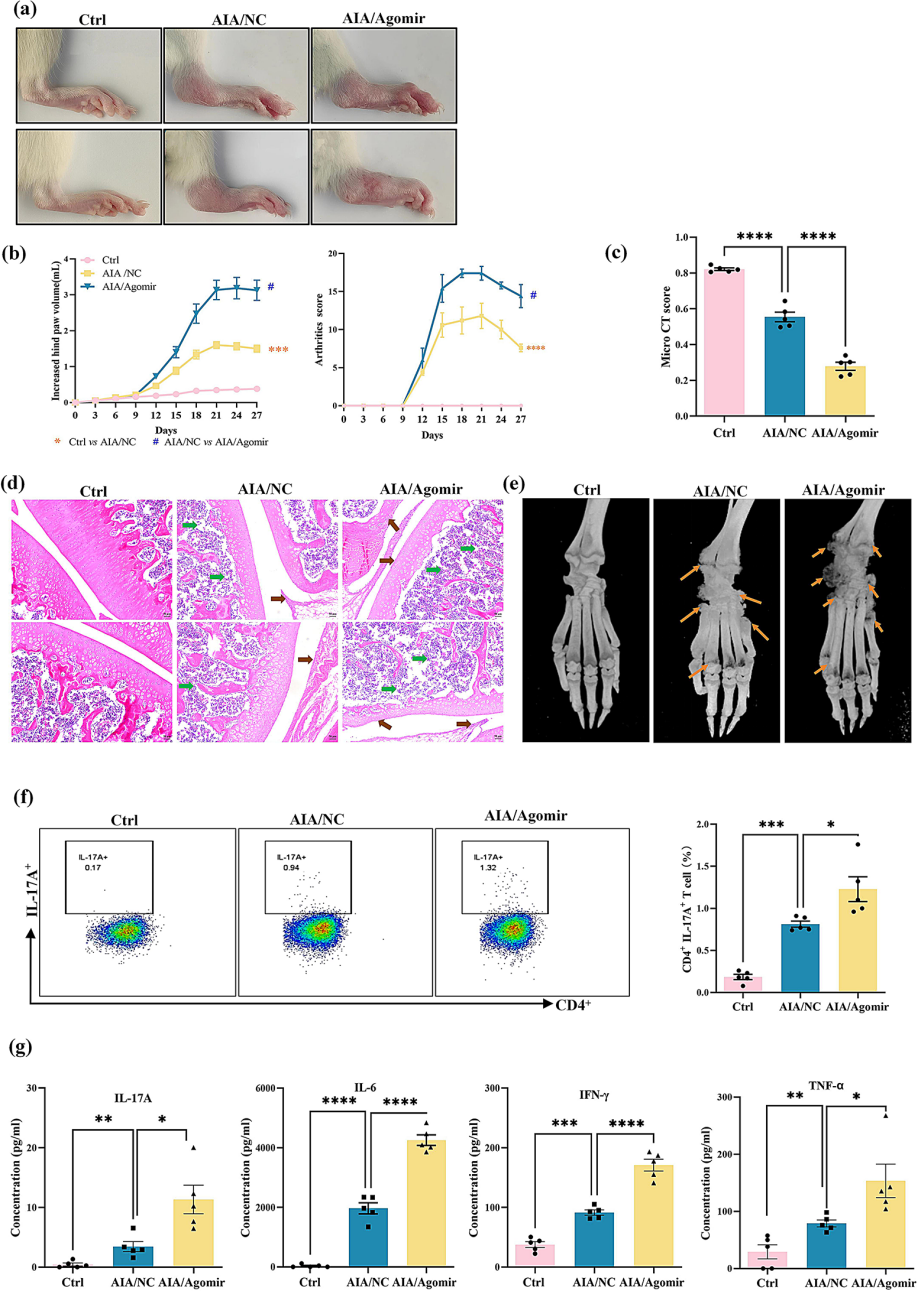
Effect of the miR-542-5p agomir on the clinical phenotype and regulation of the Th17 cells in male AIA rats. (**a**) Representative images of the hind paws of the male AIA rats treated with miR-542-5p agomir on day 27. (**b**) Changes in the hind paw volume and arthritis score of the male AIA rats treated with miR-542-5p agomir after 27 days. The data were collected at an interval of 3 days and are expressed as the mean ± SEM, *n* = 5. ^#^*P* < 0.05,^***^P < 0.001,^****^P < 0.0001, *t* test. (**c**) Micro-CT score of AIA rats. The data are expressed as the mean ± SEM (*n* = 5). ^****^P < 0.0001, *vs. the* AIA/NC group, *t* test. (**d**) Representative images of H&E-stained knee tissues from each group (magnification, 50×). Green arrows indicate bone destruction, and brown arrows indicate proliferating synovial tissues. (**e**) Representative images of 3D reconstructions of micro-CT scans of rat hind limbs. The yellow arrows indicate bone erosion. (**f**) Representative flow cytometric images illustrating the percentages of IL-17 A^+^ T cells among CD4^+^ lymphocytes. The bar chart displays the proportions of Th17 cells among CD4^+^ lymphocytes. The data are expressed as the means ± SEMs (*n* = 5). Statistical significance was assessed using a *t* test; ^*^P < 0.05 and ^***^P < 0.001 indicate significant differences compared with the AIA/NC group. (**g**) The concentration of pro-inflammatory cytokines in the serum of male AIA rats determined by a LEGENDplex™ Rat Inflammatory Panel. The results are expressed as the means ± SEMs (*n* = 5). Statistical significance was determined using a *t* test, with ^*^P < 0.05, ^**^P < 0.01, ^***^P < 0.001, and ^****^P < 0.0001 indicating significant differences compared to the AIA/NC group

In light of the aforementioned findings, a “reverse” experiment was carried out in male AIA rats, with miR-542-5p upregulated via tail vein injection of a miR-542-5p agomir. Compared with those in the AIA/NC group, a higher level of miR-542-5p was found in AIA/agomir group (Fig. S1c). Consistently, increasing miR-542-5p level led to a more pronounced arthritic phenotype in AIA rats (Fig. 6a-e). Flow cytometric analysis clarified that miR-542-5p upregulation further increased the proportion of Th17 cells and IL-17 cytokines (Fig. 6f-g). Additionally, other proinflammatory cytokines were significantly upregulated in the AIA/NC group and even more in the agomir-treated AIA group (Fig. 6g). The immune organs index was significantly increased in AIA rats after up-regulating miR-542-5p (Fig. S1d).

Taken together, these evidences suggest that miR-542-5p exhibits a potent immuno-inflammatory effect on the gender of individuals with RA pathogenesis. Suppression of miR-542-5p expression in female rats alleviates the clinical phenotype, reduces the proportion of Th17 cells, and decreases the production of immuno-inflammatory factors. However, upregulation of miR-542-5p in males exacerbates the arthritic symptoms.

### MiR-542-5p promotes the polarization of Th17 cells

**Fig. 7:**
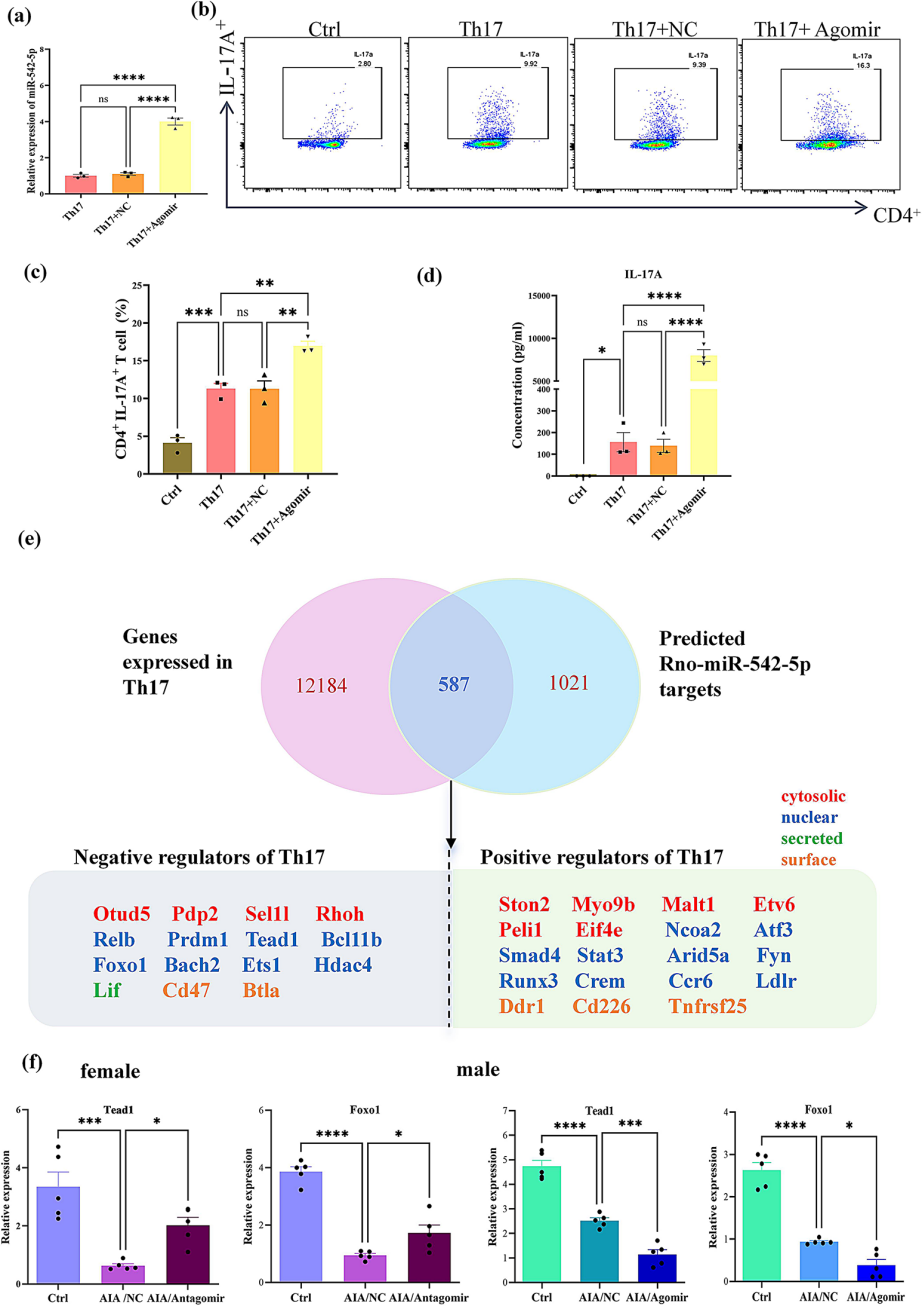
MiR-542-5p promotes the differentiation of Th17 cells *in vitro.* (**a**) The expression level of miR-542-5p in primary naive CD4^+^ T cells from male AIA rats. The fold change values were quantitatively determined by the 2^−ΔΔCt^ method for each sample after RT-PCR detection. Endogenous RNU6 was used for normalization. The data are shown as the means **±** SEMs (*n* = 3), ^****^p < 0.0001, *t* test. (**b**) Flow cytometry analysis shows the percentage of IL-17 A^+^ T cells among CD4^+^ T gate cells. (**c**) Bar chart showing the proportion of IL-17 A^+^ T cells among CD4^+^ lymphocytes. The data are shown as the means **±** SEMs (*n* = 3), ^**^P < 0.01, ^***^P < 0.001, *t* test. (**d**) Changes in the IL-17 A concentration in the supernatant of polarized CD4^+^ T cells. The data are shown as the means **±** SEMs (*n* = 3), ^*^P < 0.05, ^****^P < 0.0001, *t* test. (**e**) MiR-542-5p regulates Th17 differentiation by targeting Th17 regulators. Thirty-four miR-542-5p target candidates that regulate Th17 differentiation were identified, 15 of which are negative regulators and 19 of which are positive regulators. To select genes expressed in Th17 cells, a cutoff of average counts per million mapped reads (CPM) > 1 was applied to the transcriptome data of three Th17 single-positive RORγt + samples (GSM7111925, GSM7111926 and GSM7111927) within a mouse RNA-seq dataset (GSE227988) retrieved from the GEO database. Target Scan 8.0 was used for the prediction of Rno-miR-542-5p target genes, with “species” selected as “rat”. Negative and positive regulators of Th17 were identified from these miR-542-5p target candidates by a PubMed search for literature using the query “Th17 AND (Gene1 OR Gene 2 OR… OR Gene X)”. Gene names were concatenated with “OR” to compile the query. The localization of the gene products is represented by color, as shown in the figure legends, and was based on information deposited in the UniProt database. (**f**) Gender-based assessment of negative regulators of Th17 differentiation. The relative mRNA levels of Foxo1 and Tead1 were evaluated using RT-PCR, and the values were normalized to those of the internal control, β-actin. The data are shown as the means **±** SEMs (*n* = 5), ^*^P < 0.05, ^***^P < 0.001, ^****^P < 0.0001, *t* test

According to the findings in animal experiments, we also assessed the potential influence of miR-542-5p on Th17 cell polarization in vitro. As illustrated in Fig. 7a, following 48 h of transfection, CD4 ^+^ T cells in the agomir group exhibited significantly higher miR-542-5p expression compared to the Th17 and Th17 + NC groups. After 72 h of Th17 polarization, the proportion of Th17 cells within the CD4^+^ T-cell population increased significantly in both the Th17 and Th17 + NC groups compared with the control group, with the increase being more pronounced in the miR-542-5p overexpression group (Th17 + Agomir) (Fig. 7b-c). In addition, as depicted in Fig. 7d, the IL-17 A concentration was significantly elevated in the Th17-polarized control group, with an even greater increase observed in the agomir-treated group. Collectively, these observations suggest that miR-542-5p acts as a promoter of Th17 cell differentiation.

Subsequently, regulators of Th17 differentiation were identified through bioinformatic analysis. As depicted in Figs. 7e and 15 negative regulatory factors and 19 positive regulatory factors were identified. To further the mechanism underlying miR-542-5p-mediated Th17 differentiation, negative regulatory genes were validated *via* RT‒PCR. As shown in Fig. 7f, the expressions of these two genes were lower in AIA rats than in healthy controls (other negative regulatory factors’s expression were shown in Fig. S1e). When miR-542-5p expression was inhibited by antagomir in female AIA rats, both FOXO1 and TEAD1 expressions increased to a certain degree. In contrast, when miR-542-5p was upregulated in male AIA rats, both gene expressions were further suppressed. Taken together, miR-542-5p likely promotes Th17 cell differentiation by inhibiting the expressions of FOXO1 and TEAD1 genes.

## Discussion

Gender differences significantly influence RA, manifesting in terms of disease incidence, severity of morbidity, and treatment response [33]. Studies have indicated that, in the case of RA, women experience higher incidence and disease severity compared to men [4]. This gender preference in women is primarily attributed to sex hormones and the X chromosome [5, 34]. However, the potential mechanisms for these differential outcomes between genders have not yet been determined. To our knowledge, this is the first in vivo experimental study comparing the mechanisms of gender differences in RA to examine whether X-linked miRNAs may be involved.

There is increasing evidence for the sex-specific expression of miRNAs across auto-immune diseases, in which they could have functional implications [35, 36]. For instance, in the NZB/WF1 murine model, a noticeable difference in miRNA expression between sexes has been observed [37]. Notably, female NZB/WF1 mice exhibited increased expression of the miR-182 cluster, miR-155, miR-31, and miR-148a after the onset of lupus [37]. In our study, we observed significantly elevated expression of miR-542-5p in the RA population, with notable gender differences in its expression. In vivo experiments confirmed that upregulation of miR-542-5p accelerates the phenotype of RA. These results suggest that miR-542-5p plays a role in the pathogenesis of RA, possibly contributing to the gender bias observed in the disease.

Immunological differences across genders have been well-documented, contributing to variations in the clinical manifestations of infectious diseases, autoimmune disorders, and malignancies between the genders [38]. Differences in clinical phenotypes between female and male rats have been reported in collagen-induced arthritis (CIA) models [39]. Our study further focused on the AIA model to explore gender differences. Interestingly, in the AIA model, we also found significant gender differences, with female rats showing more severe arthritic symptoms. Recent studies have revealed that compared with male rats, female rats presented a more severe pathogenesis of CIA with a greater frequency of pathogenic T cells [40]. These findings elucidate the mechanism by which T lymphocyte cells play a role in gender differences in RA. Our data are consistent with the trends reported in the CIA rat model of arthritis, which showed a greater immune response and a higher proportion of pathogenic T cells (Th17) in female rats. In addition, our study revealed that the intensity of the immune response was greater in female AIA rats than in male control rats, which may be associated with the higher expression of miR-542-5p in female AIA rats. This finding provides a new molecular-level explanation for the increased prevalence of females in RA patients, and suggests that miR-542-5p and Th17 cells may be important factors mediating this gender difference.

Recently, studies have shown that X-linked miRNAs can participate in biological processes across genders [41]. For instance, a higher expression of miR-223 in female mice relative to male mice helps female mice better fight group B Streptococcus infection by promoting macrophage polarization [42]. In addition, the expression of miR-548am-5p in human XX (female) primary dermal fibroblasts (XX DFs) was higher than in XY (male) ones, and high expression levels of miR-548am-5p can protect XX DFs from apoptotic stimuli [41]. On the basis of our previous findings, we modulated miR-542-5p expression in a sex-dependent manner to further investigate the potential contribution of X chromosome-linked miR-542-5p to sex bias in the AIA model. The results showed that specific inhibition of miR-542-5p with an antagomir in female rats resulted in a suppressed RA clinical phenotype, while specific upregulation of miR-542-5p with an agomir in male rats exacerbated the RA clinical phenotype. Moreover, in this study, we analyzed the expansion of immune Th17 cells and found a significant decrease in Th17 cell proportion in antagomiR-542-5p-treated female rats. The opposite result was observed in the agomir-treated male rats. These findings suggest that the regulation of gender differences by miR-542-5p may be associated with the regulation of Th17 differentiation.

MiRNAs are essential regulators of immune cell differentiation and function, including the differentiation of Th17 cells [43]. For instance, miR-223 has been identified as a key regulator of chemokine signaling, enhancing Th17 differentiation while suppressing Treg differentiation [44]. These functions underscore its critical role in maintaining the balance between Tregs and Th17 cells, which is essential for Treg/Th17 homeostasis. Furthermore, research on SLE has shown that miR-542-5p exacerbates disease progression *via* the promotion of Th17 differentiation [24]. Similarly, our in vitro studies confirmed the ability of miR-542-5p to promote Th17 differentiation. Accordingly, we speculate that the increase in Th17 differentiation induced by elevated miR-542-5p leads to the more severe symptoms observed in female AIA rats than in their male counterparts. Notably, similar to the action of miR-542-5p, another mature strand miR-542-3p, derived from the same precursor miR-542, can target and inhibit foxp3 [45], a key transcription factor of Treg cells [45]. Taken together, two mature strands of miR-542 (miR-542-5p and miR-542-3p) cooperate with each other in RA by separately targeting different sides of the Treg/Th17 balance, and this phenomenon may also cause gender differences in the regulatory process.

The polarization of Th17 cells is a complex process involving the participation of numerous genes. In general, TEA domain transcription factor 1 (TEAD1) inhibits Th17 cells by antagonizing the transcriptional coactivator of activation (TAZ) [46]. Studies have confirmed that decreased forkhead box O1 (FOXO1) in SLE promotes Th17 differentiation and accelerates SLE progression [47]. Notably, both FOXO1 and TEAD1 are target genes of miR-542-5p [48]. Concomitantly, we found that their expressions were elevated when miR-542-5p expression was downregulated to suppress the Th17 differentiation in female AIA rats. In contrast, when miR-542-5p expression was upregulated in male AIA rats, the proportion of Th17 differentiation was increased, and the expression of both FOXO1 and TEAD1 was decreased. These findings suggest that the regulation of Th17 differentiation by miR-542-5p may be achieved *via* the regulations of FOXO1 and TEAD1.

Existing studies have suggested that gender-differentiated expression of X-linked genes caused by XCI may contribute to gender differences in autoimmune diseases [11]. In this context, our study tentatively confirm that the female preference in RA may be related to the differential miR-542-5p expression between male and female induced by XCI escape, as well as the regulation of Th17 cell differentiation by miR-542-5p. This provides novel insight into not only the understanding of gender-specific differences in RA and autoimmune diseases, but also the development of targeted therapeutic and preventive strategies for gender-specific conditions.

## Electronic supplementary material

Below is the link to the electronic supplementary material.

Supplementary Material 1

## Data Availability

Data are available upon reasonable request. The data are available upon reasonable request via the corresponding author.

## Abbreviations

MiRNAs: MicroRNAs
RA: Rheumatoid arthritis
AIA: Adjuvant-Induced Arthritis
XCI: X chromosome inactivation
RAFLS: Rheumatoid arthritis fibroblast like synoviocytes
OA: Osteoarthritis
SLE: Systemic Lupus Erythematosus
PBMC: Peripheral blood mononuclear cells
PFA: Paraformaldehyde
H&E: Hematoxylin & Eosin
TEAD1: TEA domain transcription factor 1
TAZ: Transcriptional coactivator of activation
FOXO1: Forkhead box O1
