# Supplementary material for: X-chromosome-linked miR-542-5p as a key regulator of sex disparity in rats with adjuvant-induced arthritis by promoting Th17 differentiation
Source: Biomark Res. 2025 Mar 1;13:36. doi: 10.1186/s40364-025-00741-x (PMC11872315; doi:10.1186/s40364-025-00741-x)
Supplement: Supplementary file 1 — Supplementary Material 1 [file 40364_2025_741_MOESM1_ESM.docx]

**Supplementary Material**

**(d)**

**(e)**

**(b)**

**(c)**

**(a)**

Figure S1 (a)In female rats, miR-542-5p expression in the AIA/anta-miR-542-5p group compared with the AIA group. The expression level of miR-542-5p in peripheral blood was quantitatively measured using real-time PCR. The miRNA expression data were normalized to the reference gene RNU6 by employing the 2^-ΔΔCT^ method for relative quantification. Statistical analysis revealed a significant difference (^****^*P*< 0.0001, *t* test) between the experimental groups. (b) The change of thymus and spleen index of female rats. The index of spleen and thymus was expressed as the ratio of wet weight of spleen and thymus to body weight (mg/g). The data are expressed as mean ± SEM (n=5). ^*^ *P*< 0.05, ^***^*P*< 0.001 *VS* the AIA control group. (c) In male rats, miR-542-5p expression in the AIA/ago-miR-542-5p group compared with the AIA group,^***^*P*< 0.001 *t* test.(d)The change of thymus and spleen index of male rats. The data are expressed as mean ± SEM (n=5). ^***^*P*< 0.01 *VS* the AIA control group. (e) Expression tendency of other Th17 negative regulatory factors based on bioinformatics analysis. The relative mRNA levels of them were evaluated using RT-PCR.
